# Sensorineural deafness in purebred white Devon Rex cats

**DOI:** 10.1111/jvim.17007

**Published:** 2024-02-08

**Authors:** Annemarie Kortas, Andrzej Pomianowski, Malgorzata Kolecka, Liliana Rytel

**Affiliations:** ^1^ Department of Internal Diseases with Clinic, Faculty of Veterinary Medicine University of Warmia and Mazury Olsztyn Poland; ^2^ Neurology and Neurosurgery Department Small Animal Clinic Kalbach Frankfurt Germany

**Keywords:** blue eyes, brainstem auditory evoked potentials, feline, hearing

## Abstract

**Background:**

Data regarding congenital sensorineural deafness (CSD) in client‐owned, white Devon Rex cats is limited because most of the information on this disease comes from experiments on mixed‐breed cats.

**Objectives:**

Provide data on the occurrence of CSD in a population of client‐owned purebred white Devon Rex cats.

**Animals:**

Forty client‐owned, purebred, white Devon Rex cats examined at 2 different facilities. Median age of the examined cats was 19 weeks.

**Methods:**

Hearing status was defined by use of brainstem auditory evoked responses.

**Results:**

The occurrence of sensorineural deafness in the studied population of Devon Rex cats was estimated at 10%. Unilateral and bilateral deafness occurred equally often, with 2 individuals having each (ie, 5.0%). No association between the occurrence of CSD and sex could be found, *χ* ^2^ (1, n = 40) = 0.001 (*P* > .99). No association between blue irises and deafness was noted in the studied population, *χ* ^2^ (1, n = 40) < 0.01 (*P* > .99).

**Conclusions:**

The occurrence of CSD in a population of client‐owned, white Devon Rex cats was found to be lower compared with data obtained in previously conducted studies of deafness in purebred cats. In the studied population of Devon Rex cats, no association between blue irises and CSD was found.

**Abbreviations**
BAER: brainstem auditory evoked responses
CSD: congenital sensorineural deafness
FERV1: feline endogenous retrovirus

## 1 INTRODUCTION

Congenital sensorineural deafness (CSD) in white cats is a hereditary condition, but its mechanism of inheritance is yet to be determined.^1^, ^2^ Cats with blue irises are known to be predisposed to CSD, which is characterized by deformities and loss of function of structures located in the inner ear, leading to hearing loss.^2^, ^3^, ^4^, ^5^ Most of the data describing the pathology of the disease was obtained from studies of mixed‐breed cats in experimental colonies, and such data are not applicable to CSD in purebred cats.^3^, ^4^, ^5^ To our knowledge, only 3 previous studies have provided data on CSD in purebred cats. However, these studies differ significantly in the number of examined individuals when compared to studies regarding CSD in dogs.^6^, ^7^, ^8^

Hereditary sensorineural deafness can manifest as a unilateral or bilateral hearing disorder and develops in the immediate postnatal days when cochlear development is still occurring, causing cats to be deaf from the first weeks of life.^1^, ^2^ Pathological changes begin with the degeneration of the stria vascularis, which leads to changes and loss of functional capacity of other elements of the cochlea in the inner ear, including Reissner's membrane.^2^, ^4^, ^9^, ^10^ The available literature indicates that degeneration of the stria vascularis is associated with the lack of functioning melanocytes responsible for maintaining a high concentration of potassium ions in the endolymph, which is necessary for the generation of an electrical potential responsible for converting external stimuli into electrical impulses.^11^, ^12^, ^13^, ^14^ The lack of melanocytes in white cats is associated with the presence of a dominant white gene (W) affecting the maturation and distribution of melanoblasts, but the exact mechanism of its inheritance has not been discovered yet.^2^, ^15^, ^16^ Previous studies provide data that supports the hypothesis that CSD is associated with a mutation of the KIT gene located within the white spotting locus (W), associated with the insertion of genetic material of the feline endogenous retrovirus (FEV1) within intron 1 of this pleiotropic and triallelic gene.^1^, ^17^, ^18^, ^19^

The only nonsubjective method available to assess hearing status in animals is analysis of results obtained during examination of brainstem auditory evoked responses (BAER) using specialized electrodiagnostic equipment.^20^, ^21^, ^22^

Our objective was to provide data on the occurrence of CSD associated with white pigmentation in a population of client‐owned Devon Rex cats.

## 2 MATERIALS AND METHODS

### 2.1 Animals

The study included 40 client‐owned white purebred Devon Rex cats. Twenty‐nine cats were examined prospectively between March 2021 and June 2023 at the Department of Internal Diseases at the Clinic of the Faculty of Veterinary Medicine of the University of Warmia and Mazury in Olsztyn, Poland as a part of a screening program to exclude deaf individuals from breeding. The remaining 11 cats were examined between November 2021 and September 2022 at a private veterinary practice located in southwestern Poland, that offered hearing testing using brainstem evoked responses. Results of the BAER examination was made available for scientific purposes after obtaining consent of the owners and were analyzed retrospectively. In accordance with Polish law, procedures undertaken as a part of a veterinary practice do not require the consent of the local ethical committee for experiments on animals.

All animals included in the study were qualified for examination using brainstem auditory evoked responses, which was preceded by a detailed interview with the animal owner and basic clinical and neurological examinations. To qualify for the study, animals had to meet the following criteria: pedigree documents confirming purebred status, >8 weeks of age; no record of ear diseases or use of potentially ototoxic drugs, and absence of ongoing disease on physical and neurological examinations.^23^ The following data had been collected from the examined Devon Rex cats: coat color, iris color, age, sex, and BAER examination results. When analyzing the relationships of the individuals, only 2 female cats included in the study were siblings from 1 litter.

### 2.2 Methods

A hearing test using BAER was performed in all animals in which the clinical and neurological examinations did not show any abnormalities. The BAER test was conducted using electrodiagnostic equipment of identical specification (Viking Quest; Nicolet Biomedical Inc, WI, USA) and the same procedure protocol was followed in both facilities. The animals were premedicated with medetomidine hydrochloride (Domitor R, Orion Corporacion Orionintie 1FIN—02200 Espoo Finland) administered IM at a dosage of 50‐150 μg/kg body weight. The animals were placed on a wooden table equipped with a rubber insulating mat and were kept in sternal recumbency throughout the entire examination. Four disposable steel needle electrodes (VIASYS Healthcare, Neurocare Group, Madison, WI USA) were placed SC in the following order and localization: (1) point Cz (at the top of the head)—recording electrode; (2) on the middle of the neck—ground electrode; and (3) over the temporal bones at the level of the mastoid processes on both sides—reference electrodes. Before placing the electrodes, the skin was disinfected using an alcohol solution (RRK‐12, CHEMPUR, Poland). To obtain results, earphones, through which the stimulus was transmitted in the form of clicks generated by electrodiagnostic equipment, were placed in the external auditory canal on the left and right sides. The left ear was examined with a click intensity at 90 dB normalized hearing level (dB nHL) before the right side. To avoid crossover stimulation while the acoustic clicks were being transmitted to the examined ear, a masking stimulus was transmitted to the nontest ear. This masking signal, also called “white noise,” was 30 dB lower than the clicks presented to the investigated ear. Before each examination was carried out, the electrodiagnostic equipment was calibrated to ensure consistency and repeatability of results. The obtained signal was amplified 200 000 times with filter settings set at the cutoff frequencies of 100 and 3000 Hz. Recordings were acquired by averaging 500‐1000 recordings of 10 ms.^20^, ^21^ After the procedure was completed, atipamezole at a dosage of 10 μg/kg IM (Antisedan, 5 mg/mL, Orion Corporation Orionintie 1 FIN02200 Espoo Finland) was used to reverse anesthesia.

**FIGURE 1:**
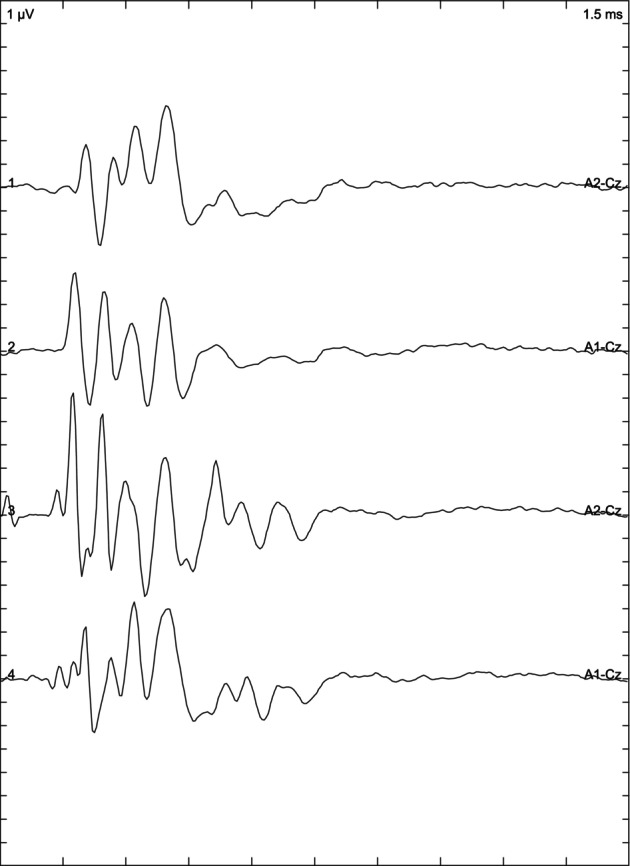
Brainstem auditory evoked response presenting bilaterally normal hearing.

**FIGURE 2:**
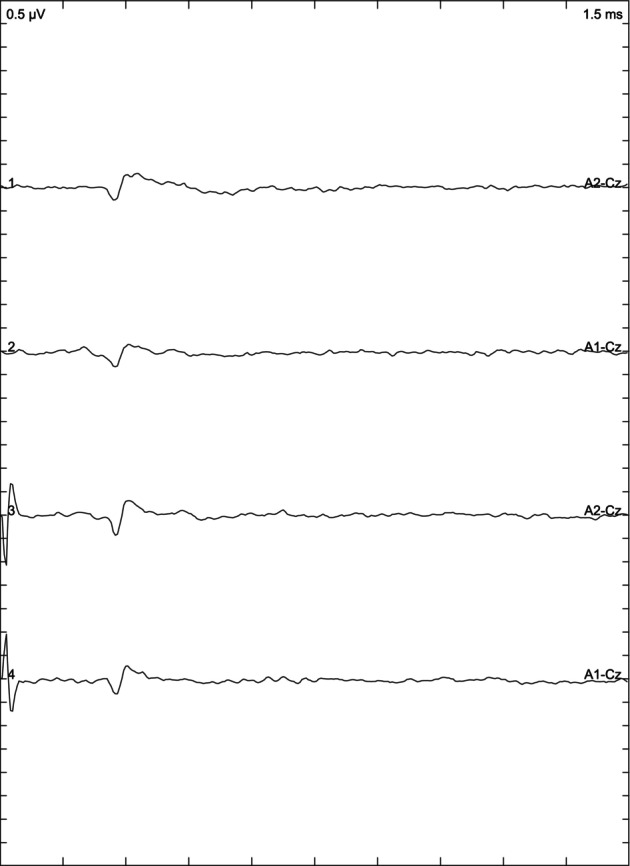
Brainstem auditory evoked response presenting bilateral deafness.

**FIGURE 3:**
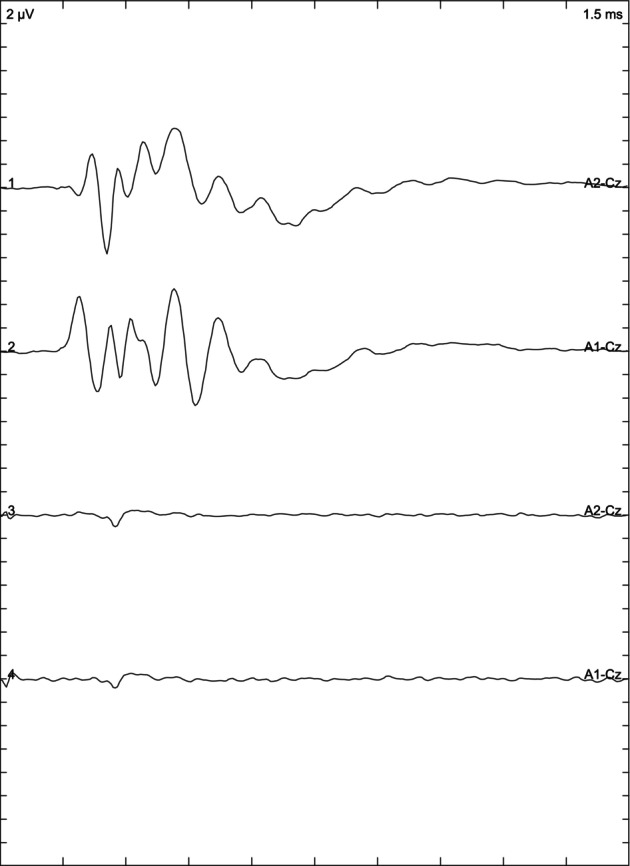
Brainstem auditory evoked response presenting right sided unilateral deafness.

Depending on the results of the BAER examination, the animals included in the study were divided into 3 groups: cats with normal hearing when the response was obtained from both ears (Figure 1), bilaterally deaf cats (Figure 2) in which no response was obtained, and unilaterally deaf cats (Figure 3) in which only 1 ear responded to the stimuli.

### 2.3 Statistical analysis

Statistical analyses were performed using R software (version 4.0.5.; R Core Team, 2021; R: Language and environment for statistical computing by R Foundation for Statistical Computing, Vienna, Austria) assuming a significance level of *α* = .05. Data are presented as count (n) as well as percentage (%) of study group. Binomial exact 95% confidence intervals (CI) for proportions were calculated as appropriate. Pearson's chi‐squared test with Yates' correction was used to determine the association between occurrence of CSD and color of the irises and, additionally, the significance between occurrence of CSD and sex of examined cats. The same test also was used to find the differences in association of CSD between unilateral and bilateral deafness. Occurrence rates regarding bilateral, unilateral, and consolidated bilateral and unilateral deafness were estimated.

## 3 RESULTS

The conducted study included 40 white purebred Devon Rex cats. The median age of all examined animals was 19 weeks. The overall occurrence of CSD in the examined population was 10.0% (4/40; 95% CI, 2.8%‐23.7%). The percentage of unilateral (2/40) and bilateral (2/40) deafness in the examined population was the same (5.0%; 95% CI, 0.6%‐16.9%) in both groups.

Hearing status was obtained for 15 male and 25 female Devon Rex cats. In the males, 2 cats were affected with CSD (13.3%; 2/15; 95% CI, 1.7%‐40.5%) and in the females, the number of individuals affected with CSD also was 2 (8.0%; 2/25; 95% CI, 0.9%‐26.0%). The differences in occurrence of CSD between male and female cats were not significant (*χ* ^2^ [1, n = 40] = 0.001; *P* > .99). Both females included in the study in which CSD was present were unilaterally deaf, whereas both males with CSD were bilaterally deaf. The comparison of percentage of unilateral and bilateral deafness showed no significant difference in the groups of cats with CSD (*χ* ^2^ [1, n = 4] < 0.01; *P* > .99).

Data regarding the color of the irises were obtained for all cats included in the study (Table 1). Eight cats had irises that were both blue, none of which was affected by deafness (ie, 0.0%; 0/8; 95% CI, 0.0%‐36.9%). Seven cats had 1 blue iris and 2 of them were affected with deafness (28.6%; 2/7; 95% CI, 3.7%‐71.0%), and among the 25 cats without blue irises, 2 cats were affected (8.0%; 2/25; 95% CI, 0.9%‐26.0%). Deafness in the group with blue irises (1 or 2) was estimated at 13.3% (2/15; 95% CI, 1.7%‐40.5%). The association between deafness and blue irises in the studied population was not significant (*χ* ^2^ [1, n = 40] < 0.01; *P* > .99). Within the group of 7 cats with just 1 blue iris 2 individuals were deaf, 1 unilaterally and 1 bilaterally (14.3%; 1/7; 95% CI, 0.4%‐57.9%) in both groups.

**TABLE 1:** Hearing status of examined cats according to the color of the irises.

Color of the irises	Number of examined cats	Cats with unilateral or bilateral CSD	Cats with unilateral CSD	Cats with bilateral CSD
Cats with 2 blue irises	8	0	0	0
Cats with 1 blue iris	7	2	1	1
Cats with both irises other than blue	25	2	1	1
Total	40	4	2	2

**FIGURE 4:**
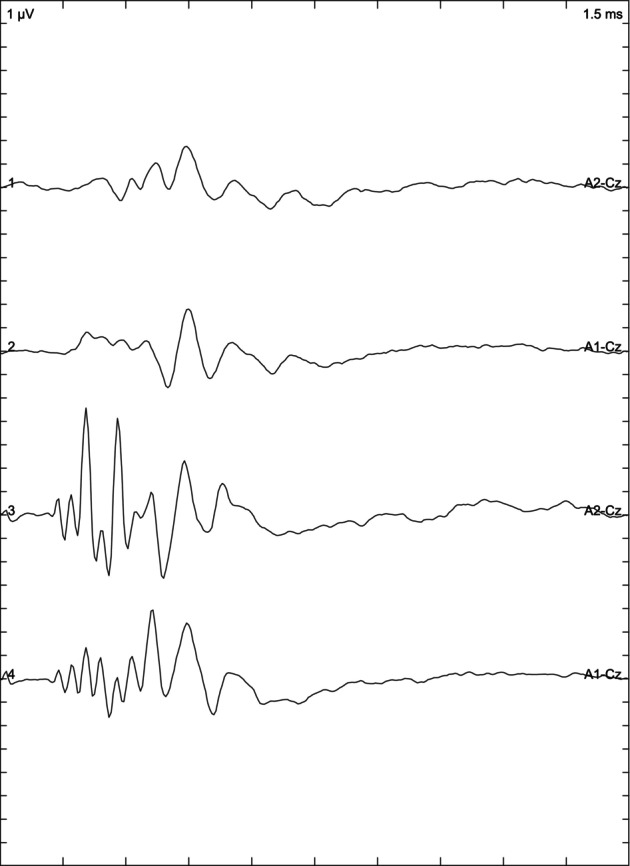
Brainstem auditory evoked response of one of the Devon Rex cats whose ears were washed on the day of the examination.

**FIGURE 5:**
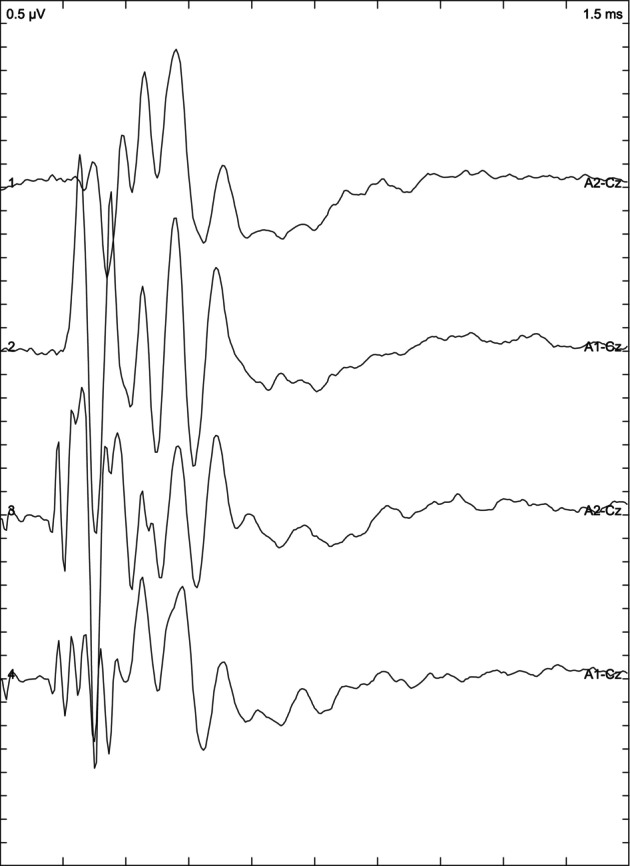
Repeated brainstem auditory evoked response (after 10 days) of one of the Devon Rex cats, whose ears were washed on the day of the first examination.

The owner of 1 cat cleaned the cat's ears on the day of the examination. In this case, the BAER examination was conducted twice: on the day the ears were cleaned (Figure 4) and 10 days later (Figure 5) to compare results and confirm if the presence of water in the external ear canal affected the results. When comparing the recordings, it was noted that the presence of water decreased the amplitude of the wave and lengthened the latency in the left ear of the examined subject when compared to the result obtained 10 days later (Figures 4 and 5).

## 4 DISCUSSION

Data regarding sensorineural deafness in purebred white cats is limited, and most of the reports relate to CSD in mixed‐breed cats and these results are not applicable to CSD in purebred cats.^3^, ^4^, ^5^ The majority of previous studies focused on describing the pathological changes that occur in the cochlea of the inner ear in white cats with CSD and the association between CSD and the presence of blue irises.^4^, ^5^, ^10^ Current information about sensorineural deafness in purebred white cats is derived from 3 studies that focused mainly on describing the prevalence of CSD.^1^, ^6^, ^7^, ^8^ Data on the prevalence of CSD in these 3 studies diverged, however, these studies included different numbers of cats of particular breeds and differed in the total number of animals examined.

Our aim was to determine the occurrence of CSD in a population of white purebred Devon Rex cats as well as to determine whether an association existed between the occurrence of CSD and the presence of blue irises among the examined individuals. For a more accurate assessment of CSD in Devon Rex cats, the association between the occurrence of CSD and sex, as well as differences in the occurrence of unilateral and bilateral deafness, were determined.

The study involved 40 completely white Devon Rex cats and the overall occurrence of CSD was estimated at 10% (4/40; 95% CI, 2.8%‐23.7%). In the previous studies, the overall prevalence of CSD in white cats was 20.2% (17/84),^6^ 30.3% (40/132)^7^ and 16.7% (12/72; 95% CI, 8.9%‐23.3%),^8^ but these results are not completely applicable to CSD in Devon Rex cats because the percentage was derived from the total number of examined white animals of different breeds. The numbers of white Devon Rex cats examined in these 3 studies were as follows: 1 (6), 12 (7), and 6 (8). The first study on the occurrence of CSD in purebred cats included 84 cats of different breeds of which only 1 cat was a Devon Rex. One cat was diagnosed as healthy but information regarding the color of its irises was not provided. Additionally, the color of the irises in this study was only determined in 55 individuals.^6^ In a study conducted less than a decade later that focused on the prevalence of sensorineural deafness in a population of purebred cats in the United Kingdom, 132 white cats of 8 different breeds were examined, of which 40 individuals were affected with unilateral or bilateral deafness. Twelve white Devon Rex cats were included in this study and 2 individuals were diagnosed with CSD (16.7%; 2/12). Unfortunately, it was not specified in that study whether cats with CSD had blue irises or not, only the total number of cats with blue irises with CSD for all breeds was provided.^7^ The next study, as well as the 2 previous studies, was limited to a small number of Devon Rex cats (n = 6), among which only 1 had CSD and did not have blue irises.^8^ The small number of Devon Rex cats in the mentioned studies makes it impractical to compare the prevalence and occurrence of deafness with the results obtained in our study. Presented figures regarding the size of the studied populations indicate the need to conduct further research on a larger number of animals. Currently, data on the prevalence of CSD in cats differs quantitatively from the available data on CSD in various breeds of dogs.^24^, ^25^, ^26^ Studies regarding CSD in dogs include 100's to 1000's of animals, and thus the presented data have greater statistical value. We collected hearing status results and phenotypic data from only 40 cats, but we believe cooperation of more facilities with electrodiagnostic equipment would enable collection of sufficient data to match that collected for dogs with CSD.

In our study, the association between deafness and blue irises was not significant (*χ* ^2^ [1, n = 40] < 0.01; *P* > .99) which is consistent with a previous study (*χ* ^2^ [1, n = 72] < 0.01; *P* = .91),^8^ but does not coincide with results of other studies (*P* = .04)^6^ and (*χ* ^2^ > 3.85; *P* ≤ .05).^7^ Although our results differ from data published previously, which may result from the different number of individuals and the variety of breeds, no other studies have described CSD in purebred cats. Differences in the obtained results may be a consequence of a different genetic pool as well as other reproductive strategies in the studied populations. In the study of 8 cats with 2 blue irises, neither had CSD, whereas 2 cats with 1 blue iris and 2 cats with both irises of color other than blue were diagnosed with CSD. The occurrence of deafness in cats with at least 1 blue iris was estimated at 13.3% (2/15; 95% CI, 1.7%‐40.5%) but in our study, there was no difference in association between the presence of CSD in cats with 1 or 2 blue irises, which confirms the data obtained in previous studies (*χ* ^2^ [1, n = 15] = 0.74; *P* = 0.39).^6^, ^7^, ^8^

In a previous study it was noteworthy that a similar number of cases were examined as were examined by us.^7^ The prevalence of CSD in the Norwegian Forrest cats in the previous study was found to be 43.9% (18/40), which is significantly higher than the 10% (4/40) occurrence of CSD in Devon Rex cats in our study.^7^ The indicated dissimilarity in these 2 cat populations may be caused by differences such as reproductive strategies or randomness characterizing clinical trials but also may indicate that Devon Rex cats are less likely to have CSD compared with Norwegian Forest cats. Substantial differences are found in the prevalence of CSD among dog breeds with white pigmentation, and it is possible that similar differences exist among cat breeds.^24^, ^25^, ^26^ A previous study that used logistic regression data found that Norwegian Forest cats were significantly associated with the presence of CSD compared with the remainder of the cats grouped together as non‐Norwegian cats in the examined population.^7^ Data presented in that study indicated that cats with at least 1 blue iris were 3.2 times more likely to have CSD compared with Norwegian Forest cats with irises of color other than blue, whereas in our study no association between CSD and the presence of at least 1 blue iris could be found.^7^ Our study's limited sample size of cats could be a reason for the lack of association that was identified.

In our study, CSD was diagnosed in 2 of 15 examined males (ie, 13.3%; 2/15; 95% CI, 1.7%‐40.5%) and in 2 of the 25 examined females, (ie, 8.0%; 2/25; 95% CI, 0.9%‐26.0%). No association was found between the occurrence of CSD in Devon Rex cats and sex (*χ* ^2^ [1, n = 40] = .001; *P* > .99), which is consistent with previously reported data regarding a higher number of breeds.^6^, ^7^, ^8^ In our study, both Devon Rex females with CSD were unilaterally deaf, and similar findings were described in a previously conducted study.^8^

In our research, of 40 Devon Rex cats, 2 cats were unilaterally deaf and the remaining 2 cats had bilateral CSD. The percentage of unilateral and bilateral deafness in the examined population therefore was the same and estimated at 5.0% (95% CI, 0.6%‐16.9%) in both groups. In the cats with deafness, the comparison of percentage of unilateral and bilateral deafness showed no significant difference (*χ* ^2^ [1, n = 4] < 0.01; *P* > .99). Data on differences in the occurrence of unilateral and bilateral deafness are extremely limited and inconsistent in the literature. Two previous studies reported the occurrence of unilateral and bilateral deafness at a similar percentage,^6^, ^7^ whereas another independent study provided data that, in the surveyed population, unilateral deafness was twice as common as bilateral deafness.^8^ Because of the lack of conclusive data, further research on the occurrence of these types of deafness in the population of cats with CSD is required. In addition, the fact that the exact mechanisms of CSD inheritance, and in particular the genetic basis of the occurrence of unilateral and bilateral deafness, have not yet been described, indicates the need to conduct research on a larger number of cats affected by CSD.

In conclusion, our results suggest a lower occurrence of CSD in white Devon Rex cats when compared to previously conducted studies on the prevalence of CSD in purebred white cats.^6^, ^7^, ^8^ Another difference when comparing our results to the available literature is the lack of association between the occurrence of deafness and the presence of blue irises.^6^, ^7^ These dissimilarities may result from numerous differences between the compared populations, and also may be caused by the limited number of animals examined in the studied groups. However, the contrast between the occurrence of CSD and its association with blue irises in Devon Rex and Norwegian Forest cats is particularly noteworthy. The indicated dissimilarities justify subsequent research into the occurrence of CSD in particular breeds of cats in which the white (W) gene is present.^27^ Breeder awareness about CSD and the interest in decreasing or eliminating the disease from the population of purebred cats has increased in Poland in recent years, as confirmed by the resolution of 01/01/2021 (Resolution of the General Meeting of Members of the Polish Felinological Federation “Felis Polonia” [FPL]).^28^ We believe that the introduced resolution may contribute to discovery of yet undescribed mechanisms of inheritance and prevalence rates, because it indicates the need to perform hearing examination with the use of BAER in all white cats intended for breeding.

## CONFLICT OF INTEREST DECLARATION

Authors declare no conflict of interest.

## OFF‐LABEL ANTIMICROBIAL DECLARATION

Authors declare no off‐label use of antimicrobials.

## INSTITUTIONAL ANIMAL CARE AND USE COMMITTEE (IACUC) OR OTHER APPROVAL DECLARATION

Authors declare no IACUC or other approval was needed.

## HUMAN ETHICS APPROVAL DECLARATION

Authors declare human ethics approval was not needed for this study.
